# Inverse association of circulating kallikrein-related peptidase 7 with renal function and mortality risk in patients with chronic kidney disease

**DOI:** 10.3389/fendo.2026.1804956

**Published:** 2026-07-07

**Authors:** Robin Schürfeld, Fabian Baalmann, Ekaterine Baratashvili, Benjamin Sandner, Anette Bachmann, Juliane Weiner, Marleen Würfel, Ralph Wendt, Martin Haussmann, Ingolf Bast, Joachim Beige, Joanna Kosacka, Nora Klöting, Knut Krohn, Toralf Kirsten, Ming-Zhi Zhang, Raymond C. Harris, Berend Isermann, Peter Kovacs, Matthias Blüher, Michael Stumvoll, Anke Tönjes, John T. Heiker, Thomas Ebert

**Affiliations:** 1 Medical Department III – Endocrinology, Nephrology, Rheumatology, University of Leipzig, Leipzig, Germany; 2 Department of Nephrology, Hospital St. Georg Leipzig, Leipzig, Germany; 3 Nierenzentrum Leipzig, Outpatient Care Units, Leipzig, Germany; 4 Patienten-Heimversorgung-Dialysezentrum Leipzig-Wiederitzsch, Leipzig, Germany; 5 Kuratorium for Dialysis and Kidney Transplantation, Neu-Isenburg, Germany; 6 Martin-Luther-University Halle/Wittenberg, Halle/Saale, Germany; 7 Department of Neurology, University of Leipzig, Leipzig, Germany; 8 Helmholtz Institute for Metabolic, Obesity and Vascular Research (HI-MAG) of the Helmholtz Zentrum München at the University of Leipzig and University Hospital Leipzig, Leipzig, Germany; 9 Medical Faculty, University of Leipzig, Leipzig, Germany; 10 Institute for Medical Informatics, Statistics and Epidemiology, Leipzig University, Leipzig, Germany; 11 Division of Nephrology, Department of Medicine, Vanderbilt University School of Medicine, Nashville, TN, United States; 12 Department of Medicine, Nashville Veterans Affairs Hospital, Vanderbilt University School of Medicine, Nashville, TN, United States; 13 Institute of Laboratory Medicine, Clinical Chemistry and Molecular Diagnostics (ILM), University Hospital Leipzig, Leipzig, Germany; 14 LeiCeM - Leipzig Center of Metabolism, Leipzig University, Leipzig, Germany; 15 Deutsches Zentrum für Diabetesforschung, Neuherberg, Germany

**Keywords:** adipose tissue, chronic kidney disease, dialysis, kallikrein-related peptidase 7, mortality

## Abstract

**Introduction:**

Kallikrein-related peptidase 7 (KLK7) is a protease implicated in metabolic disease and obesity. Patients with chronic kidney disease (CKD) exhibit several cardio-metabolic comorbidities and increased mortality. The goal of this study was to investigate the associations of KLK7 levels with renal function and clinical outcomes in patients with CKD.

**Methods:**

Baseline KLK7 serum levels were cross-sectionally related to renal and cardiometabolic markers in the Leipzig-CKD cohort (n=542). Longitudinal Cox Regression analyses (n=472) were performed to associate baseline circulating KLK7 concentrations and risk of major adverse renal (MARE) and cardiovascular (MACE) events, as well as all-cause mortality. Additionally, mRNA expression of *Klk7* and related genes was examined in CKD versus control mice using bulk RNA sequencing.

**Results:**

KLK7 levels were inversely associated with markers of renal function, i.e. estimated glomerular filtration rate (eGFR), and inflammation, i.e. C-reactive protein, in multivariable regression. Longitudinal analyses associated higher baseline KLK7 with lower all-cause (adjusted HR [95% CI]: 0.65 [0.49–0.86], p=0.003) and non-cardiovascular (adjusted HR [95% CI]: 0.56 [0.40–0.78], p=0.001) mortality over a median follow-up of 7.6 years. A KLK7 threshold of 1383.6 pg/ml stratified patients into low- and high-risk groups for mortality. No associations were found for MARE or MACE. In tissues of CKD and control mice, no significant changes in *Klk7* mRNA expression were found.

**Discussion:**

Circulating KLK7 associates inversely with renal function and inflammation, likely reflecting reduced renal clearance. Higher circulating KLK7 independently predicts lower all-cause mortality, whereas baseline KLK7 was not related to composite renal and CV outcomes.

## Graphical Abstract

**Figure:**
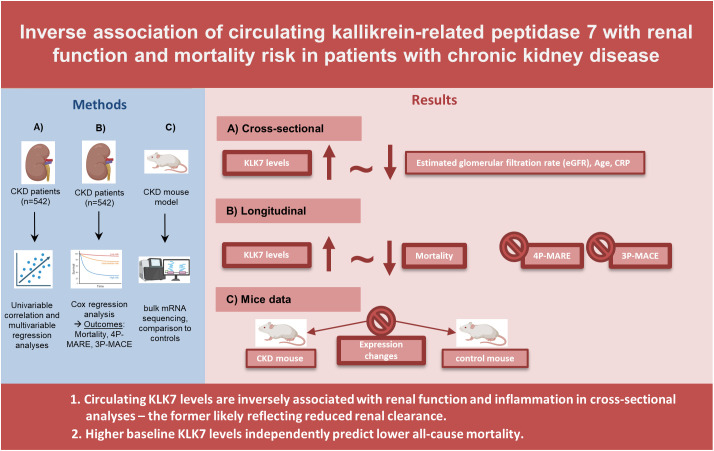

## Introduction

Chronic kidney disease (CKD) represents a significant global health challenge, responsible for an estimated 1.2 million deaths annually (1). The most frequent etiologies for CKD are diabetes and hypertension, and the disease is further driven by mechanisms, such as chronic inflammation, increased oxidative stress, and cellular aging (2, 3). These factors contribute to the high mortality rates in CKD patients, with cardiovascular (CV) causes accounting for the majority of all deaths, i.e. ~40–50% (4). The inter−related pathophysiology of CKD, obesity, and systemic inflammation is now recognized as a central component of the cardiovascular-kidney-metabolic (CKM) syndrome (5). Because most patients with CKD remain asymptomatic until advanced stages, the identification of novel biomarkers that reflect both obesity−related inflammation and renal pathology could improve early detection and patient management. Given these associations, there is a pressing need to investigate endocrine cytokines that may serve as molecular links between impaired renal function and cardiometabolic dysfunction.

Kallikrein-related peptidase 7 (KLK7) is a serine protease with multifaceted roles across several skin disorders (6, 7), cancers, and metabolic processes (8). Thus, adipose tissue-specific knockout of KLK7 in mice confers protection against high-fat diet (HFD)-induced obesity, characterized by reduced weight gain, with enhanced energy expenditure and improved insulin sensitivity, due to reduced adipose tissue inflammation (9). Similar results were observed for whole-body KLK7 knockout mice (10). Moreover, a macrophage-specific KLK7 knockout in HFD-fed mice leads to lower levels of systemic inflammation and reduced infiltration of proinflammatory macrophages into epididymal adipose tissue independent of weight gain (11). In line with these findings, serum KLK7 levels in individuals with obesity and type 2 diabetes were strongly correlated with circulating inflammatory markers (11).

Interestingly, transcriptomic analyses of human kidney tissue have shown that *KLK7* is differentially expressed in glomeruli of patients with diabetes and CKD compared to healthy controls (12). As KLK7, therefore, might be an endocrine factor linking different components of the CKM syndrome, we hypothesized that KLK7 regulation in CKD is related to cardiometabolic and mortality outcomes, and aimed 1) to investigate the associations of circulating KLK7 with cardio-kidney-metabolic markers in a cross-sectional analysis in patients with CKD; 2) to evaluate its relationship with a broad spectrum of clinically relevant renal, CV, and mortality outcomes in longitudinal analyses; and 3) to examine mRNA expression of *Klk7* and related genes in mice with type 2 diabetes, obesity, and diabetic kidney disease (DKD).

## Materials and methods

### Study population (Leipzig-CKD)

Subjects included in this cohort, as well as inclusion/exclusion criteria, have been extensively described elsewhere (13–17). In brief, 581 patients (338 men; 243 women) were recruited from the Department of Endocrinology and Nephrology at the University of Leipzig, as well as from three different outpatient Nephrology Care Units (Hospital St. Georg (Division of Nephrology), KfH Renal unit Leipzig, and outpatient Nephrology Care Units) from 2006 - 2014.

For this analysis, serum specimens were available for 542 of the 581 participants. In 2023, follow-up data were collected as recently described (14). Of this part of the original cohort, about 472 patients (87.1%) were successfully followed up after a median follow-up of 7.6 years.

The study was approved by the Ethics Committee of the University of Leipzig (Reg. No: 180-13-15072013). Prior to inclusion, informed written consent was obtained from all participants.

### Biochemical and clinical parameters

In all participants, a blood sample was drawn after an overnight fast. For patients undergoing dialysis, fasting blood samples were drawn immediately prior to dialysis treatment. eGFR was calculated using the Chronic Kidney Disease Epidemiology Collaboration (CKD-EPI) equation based on serum creatinine measurements (18). Patients were then classified into five eGFR categories (G1–G5) according to the Kidney Disease: Improving Global Outcomes (KDIGO) guidelines (19). Urine albumin (Albuwell h Albumin) and creatinine (Creatinine Companion) were quantified from spot urine samples and analyzed using commercial ELISA kits from Exocell (Philadelphia, PA) to assess uACR. Serum levels of KLK7 (ab287175, Abcam, Cambridge, UK) were measured using ELISA kits according to the manufacturer’s instructions. Routine serum parameters, including creatinine, fasting glucose (FG), fasting insulin (FI), glycated hemoglobin A1c (HbA1c), triglycerides (TG), high density lipoprotein (HDL), low density lipoprotein (LDL) cholesterol, and C-reactive protein (CRP) were measured in a certified laboratory. A FG ≥ 7.0 mmol/l or use of insulin or other glucose-lowering medication was classified as type 2 diabetes (T2D) (20).

### Longitudinal outcomes

Similar to Würfel et al. (14), major adverse renal events (MARE) were assessed as a 4-point composite (4P-MARE) consisting of incident kidney disease, worsening kidney disease, initiation of kidney replacement therapy (KRT), or death due to renal causes according to Prischl and colleagues (21). Incident kidney disease was new onset of kidney injury, indicated by eGFR <60 ml/min/1.73 m² and/or albuminuria (uACR >30 mg/g). Worsening of kidney disease referred to a sustained eGFR decline >40% and/or progression of uACR from stage A2 (30–300 mg/g) to A3 (>300 mg/g). KRT included dialysis or transplantation; renal death was mortality directly attributable to kidney disease (21).

Major adverse cardiovascular events (MACE) were a 3-point composite (3P-MACE) defined as nonfatal myocardial infarction, nonfatal stroke, or cardiovascular death classified as primary cause (with ICD-10 codes I00.xx–I99.xx) (22). Atherosclerotic cardiovascular disease (ASCVD) was defined as the presence of any of the following diseases: heart failure, coronary artery disease, acute myocardial infarction, stroke, or peripheral arterial disease (23).

### Animal study

For this study, a CKD mouse model with T2D (db/db;eNOS^-/-^ mice) was compared to nondiabetic (eNOS^-/-^) control mice as described previously (15, 24). The db/db;eNOS-/- mice model was chosen, because it most comprehensively reflects all aspects of the CKM syndrome (5), i.e. T2D, obesity, endothelial dysfunction, hypertension, dyslipidemia, and CKD. Therefore, this model closely captures the metabolic and vascular abnormalities relevant to investigating the potential role of Klk7 in the context of CKD. All animal experiments were performed in the Medical Experimental Center at the University of Leipzig and approved by the local ethics committee of the state of Saxony (Landesdirektion Leipzig; approval numbers TVV12/14, 30/19, and 16/24). Mice were kept under pathogen-free conditions with *ad libitum* access to water and food. The following tissues were harvested for mRNA isolation, each at the age of 24 weeks: Visceral (VAT), subcutaneous (SAT) and brown adipose tissue (BAT), kidney and liver. These tissues were chosen based on the established role of KLK7 in adipose tissue inflammation (11), the crucial role of the liver in the synthesis of many plasma proteins and the known expression of KLK7 in glomeruli of patients with diabetes and CKD (12).

### Bulk mRNA sequencing

Tissues from ≥five animals per group were immersed with TRIzol (Sigma-Aldrich, Merck, Darmstadt, Germany) and homogenized using a Precellys 2 homogenizer (Bertin Technologies SAS, Montigny-le-Bretonneux, France). The mRNA was isolated using the RNeasy Lipid Tissue Kit (Qiagen, Hilden, Germany). RNA quantity and quality were measured with NanoVue spectrophotometer (GE HealthCare GmbH, Düsseldorf, Germany) and Fragment Analyzer 5200 (Agilent, Waldbronn, Germany) using the High Sensitivity RNA quantification kit and Fragment Analyzer Controller Software (Agilent v3.1.0.12). Random primed library preparation was started with 150 ng of total RNA using the Watchmaker RNA library prep kit with Polaris depletion (Watchmaker Genomics, Boulder, CO). The barcoded libraries were purified and quantified using Qubit Fluorometric Quantification (ThermoFischer Scientific, Schwerte, Germany). Sequencing of 2x150 bp was performed with an Illumina NovaSeq 6000 sequencer (Illumina, San Diego, CA) at the sequencing core facility of the Faculty of Medicine at the University of Leipzig. After demultiplexing with bcl2fastq software (Illumina, v2.20) and polishing using FASTP (25) reads were mapped against the mouse reference genome (GRCm39, September 02, 2020) using hisat2 (v2.2.1) (26).

### Statistical methods

All statistical analyses were performed using R version 4.5.1 (27). Trends across eGFR categories were assessed by Jonckheere-Terpstra test for continuous variables and Cochran-Armitage test for categorial variables. For cross-sectional analyses, univariate correlations were performed using Spearman`s rank correlation method. All parameters that showed significant univariate correlations with KLK7 were included in a multivariable linear regression analysis with further adjustment for age, BMI, and sex. Prior to multivariable testing, non-normally distributed variables, as assessed by Shapiro-Wilk-test, were logarithmically transformed (lg).

For longitudinal analyses, multivariable Cox regression analyses were performed to associate baseline serum KLK7 levels with the aforementioned pre-defined outcomes. Variables included in the Cox regression models were selected based on their statistical significance in the cross-sectional analyses or biological relevance (28). The adjusted Hazard ratios (HR) with 95% confidence intervals (CI) for the whole range of KLK7 were estimated from the respective Cox model with the *Predict* function from the *rms* package. HRs of circulating KLK7 for the risk of incident endpoints are expressed per one unit difference in the log2-transformed KLK7 concentration, equivalent to a doubling of serum KLK7. The optimal KLK7 cut-off value for stratifying patients into high- and low-risk groups for mortality was determined by applying Youden’s index to the Receiver Operating Characteristic (ROC) analysis.

In all analyses, a p-value < 0.05 was considered statistically significant.

Differential mRNA expression analysis for protein-coding genes of the mouse experiments was done using the *DESeq2* package (29). For correlation analysis, read counts were normalized using the *vst* function of the *DESeq2* package (29). For mouse bulk mRNA sequencing data, FDR-adjusted p-values < 0.05 were considered significant.

### Artificial Intelligence Generated Content (AIGC) tools

Any Artificial Intelligence Generated Content (AIGC) tools such as ChatGPT and others based on large language models (LLMs) were not used in developing any portion of the manuscript.

## Results

### Baseline characteristics of the study population (n=542)

**Table 1** presents the baseline characteristics of the study population stratified by CKD categories G1-G5. Participants in more advanced CKD stages were generally older (p<0.01) and more often smokers (p<0.01), while the stratified groups did not differ in diabetes prevalence or sex distribution. Markers of glucose metabolism and insulin resistance, including FG, FI, and HbA1c progressively increased from eGFR category G1 to G4 (all p<0.01). Lipid profiles showed decreasing HDL and LDL cholesterol levels with higher CKD stages (p<0.01). In contrast, TG levels, markers of impaired renal function (creatinine, uACR) and inflammation (CRP) significantly increased with higher CKD stages (all p<0.01). Median [interquartile range] serum KLK7 levels were 1.6 [8.6] ng/ml in the total sample and increased significantly with increasing CKD categories as well (**Table 1**).

**Table 1:** Baseline characteristics of the entire study cohort, stratified by estimated glomerular filtration rate (eGFR) categories.

Variable	G1	G2	G3	G4	G5	p for trend
N	57	87	131	70	197	NA
Female (N [%])	30 (52.6%)	46 (52.9%)	43 (32.8%)	33 (47.1%)	79 (40.1%)	0.09
Diabetes (N [%])	20 (35.1%)	36 (41.4%)	59 (45.0%)	32 (45.7%)	79 (40.1%)	0.79
Current smoker (N [%])	8 (14.0%)	7 (8.0%)	10 (7.6%)	4 (5.7%)	37 (18.8%)	**0.03**
Dialysis therapy (N [%])	0 (0.0%)	0 (0.0%)	0 (0.0%)	0 (0.0%)	181 (91.9%)	**<0.01**
Age (years)	54.2 (45.4–63.8)	64.8 (55.2–69.8)	72.6 (64.0–77.3)	73.6 (67.3–80.2)	66.4 (53.5–74.6)	**<0.01**
BMI (kg/m²)	26.4 (24.0–29.8)	28.7 (26.1–31.9)	28.3 (25.6–30.9)	27.1 (24.7–30.2)	26.2 (23.4–30.4)	**<0.01**
Waist circumference (cm)	95 (87–106)	102 (93–112)	102 (97–111)	102 (94–110)	104 (93–113)	0.06
Waist-to-height ratio	0.6 (0.5–0.6)	0.6 (0.5–0.7)	0.6 (0.6–0.7)	0.6 (0.6–0.7)	0.6 (0.5–0.7)	0.09
SBP (mmHg)	129 (120–140)	130 (120–150)	135 (125–150)	140 (130–150)	130 (117–140)	0.20
DBP (mmHg)	80 (70–85)	80 (70–90)	80 (73–90)	80 (70–88)	75 (65–80)	**<0.01**
FG (mmol/l)	5.3 (4.7–6.3)	5.8 (5.0–6.9)	5.8 (5.0–7.1)	5.7 (5.0–6.6)	4.9 (4.2–6.0)	**<0.01**
FI (pmol/l)	55.6 (34.4–84.4)	71.5 (41.0–112.8)	76.9 (43.3–139.3)	76.1 (51.0–107.2)	45.5 (25.5–95.5)	**<0.01**
HbA1c (%)	5.5 (5.4–5.8)	5.7 (5.5–6.2)	5.8 (5.5–6.1)	5.8 (5.5–6.1)	5.4 (5.1–5.8)	**<0.01**
Total cholesterol (mmol/l)	5.3 (4.6–6.0)	5.5 (4.9–6.2)	5.3 (4.6–6.2)	6.0 (4.6–7.1)	4.7 (3.8–5.6)	**<0.01**
HDL cholesterol (mmol/l)	1.4 (1.2–1.8)	1.3 (1.1–1.5)	1.3 (1.1–1.6)	1.4 (1.0–1.7)	1.1 (0.9–1.4)	**<0.01**
LDL cholesterol (mmol/l)	3.3 (2.5–3.9)	3.2 (2.6–4.0)	3.0 (2.4–3.8)	3.2 (2.4–4.2)	2.7 (2.0–3.3)	**<0.01**
TG (mmol/l)	1.2 (0.9–1.6)	1.5 (1.2–2.1)	1.6 (1.2–2.3)	1.7 (1.2–2.6)	1.6 (1.1–2.2)	**<0.01**
Creatinine (μmol/l)	63 (56–72)	82 (73–97)	137 (116–159)	210 (179–241)	667 (480–877)	**<0.01**
eGFR (ml/min/1.73m^2^)	98.8 (93.9–104.9)	74.1 (68.9–81.6)	41.8 (35.7–49.6)	23.7 (20.3–26.3)	6.0 (4.4–9.0)	**<0.01**
uACR (mg/g)	7.1 (4.8–13.4)	11.6 (4.5–46.9)	22.1 (6.6–69.6)	89.1 (14.2–246.0)	207.5 (63.0–521.3)	**<0.01**
CRP (mg/l)	1.8 (0.6–4.7)	2.4 (1.2–5.0)	2.8 (1.4–4.5)	3.5 (1.5–6.2)	4.4 (1.9–12.2)	**<0.01**
KLK7 (ng/ml)	1.3 (1.0–1.7)	1.4 (1.1–1.9)	1.6 (1.2–1.9)	1.5 (1.3–1.9)	1.7 (1.2–2.5)	**<0.01**

Baseline characteristics of the entire study cohort, stratified by estimated glomerular filtration rate (eGFR) categories.

BMI, Body-mass index; CRP, C reactive protein; DBP, Diastolic blood pressure; eGFR, estimated glomerular filtration rate; FI, Fasting insulin; FG, Fasting glucose; HbA1c, glycated hemoglobin A1c; HDL, High-density lipoprotein; KLK7, Kallikrein-related peptidase 7; LDL, Low-density lipoprotein; SBP, Systolic blood pressure; TG, Triglycerides; uACR, urine albumin-creatinine ratio. Values for median (Q1 - Q3) or total number [percentage] are shown. P values for trend were assessed by Jonckheere-Terpstra test for continuous variables and Cochran Armitage test for categorial variables.Bold markers indicate statistically significant p values (p<0.05).

### Cross-sectional univariate und multivariable correlations (n=542)

In univariate correlations, KLK7 serum levels were inversely associated with age, BMI, eGFR, FG, HDL cholesterol, and CRP (all p <0.05; **Table 2**). Furthermore, serum KLK7 significantly increased with higher creatinine levels and uACR (all p <0.05; **Table 2**). To cross-sectionally identify independent predictors of circulating KLK7 concentrations, multivariable regression analyses were carried out for all parameters that significantly correlated with KLK7 levels in univariate analyses. Here, age, eGFR, and CRP were the strongest, inverse predictors of KLK7 (all p<0.05) after adjusting for sex, BMI, FG, HDL cholesterol, and uACR. In addition, KLK7 was associated with male sex and lower HDL cholesterol levels (**Table 2**). In sex-stratified analyses, age, eGFR, and CRP remained the strongest inverse predictors of KLK7 (all p<0.05) ( **Supplementary Tables 1**, **2**).

**Table 2:** Univariate correlation analyses and multivariable linear regression analysis of serum KLK7 with anthropometric parameters and markers of glucose metabolism, serum lipids, inflammation, and renal function.

Variable	Univariate correlation analyses		Multivariable linear regression analysis	
	r	p	β	p
Age (years)	**-0.247**	**<0.001**	**-0.236**	**<0.001**
Sex	**-**	**-**	**-0.117**	**0.026**
BMI (kg/m^2^)	**-0.102**	**0.018**		
Waist circumference (cm)	-0.047	n.s.	–	–
Waist-to-height ratio	**-0.121**	**0.005**	0.010	n.s.
SBP (mmHg)	-0.044	n.s.	–	–
DBP (mmHg)	0.036	n.s.	–	–
HbA1c (%)	-0.081	n.s.	–	–
FG (mmol/l)	**-0.150**	**<0.001**	0.022	n.s.
FI (pmol/l)	-0.060	n.s.	–	–
Cholesterol (mmol/l)	-0.079	n.s.	–	–
HDL cholesterol (mmol/l)	**-0.124**	**0.004**	**-0.153**	**0.007**
LDL cholesterol (mmol/l)	-0.066	n.s.	–	–
TG (mmol/l)	0.034	n.s.	–	–
Creatinine (µmol/l)	**0.267**	**<0.001**	–	–
eGFR (ml/min/1.73m²)	**-0.218**	**<0.001**	**-0.233**	**<0.001**
uACR (mg/g)	**0.147**	**0.006**	-0.012	n.s.
CRP (mg/l)	**-0.151**	**<0.001**	**-0.227**	**<0.001**

Non-parametric Spearman’s rank correlation method was used to assess univariate relationships between KLK7 and indicated markers. Multivariable regression analysis was calculated for KLK7 (lg, dependent variable) adjusted for age (lg), sex, Waist-to-height ratio, FG (lg), HDL cholesterol (lg), eGFR (lg), uACR (lg) as well as CRP (lg). Non-normally distributed variables as assessed by Shapiro-Wilk-test were logarithmically transformed prior to multivariable testing (lg). r- and p-values, as well as standardized β-coefficients and p-values, are given. Bold markers indicate significant correlations in univariate analysis or independent associations in multivariable analyses. Abbreviations are indicated in **Table 1**.

### Longitudinal associations of KLK7 serum levels with mortality, 4P-MARE, and 3P-MACE (n=472)

Follow-up data was available for 472 individuals. A total of 180 of these participants died during follow-up (CV deaths: n=49; non-CV deaths: n=131). About 148 developed a 4P-MARE endpoint. Specific renal outcomes included 25 patients with incident CKD, 118 with worsening kidney disease, 59 with novel KRT, and 8 who died due to a renal cause. CV complications during follow-up occurred in 49 (non-fatal stroke) and 28 (non-fatal myocardial infarction) individuals, respectively.

Unadjusted Cox regression analyses revealed that higher baseline log2-transformed KLK7 concentrations were significantly associated with lower all-cause mortality (HR 0.66 [95% CI: 0.53-0.84], p = 0.001), but not with 4P-MARE or 3P-MACE (Model 1, **Table 3**). As serum KLK7 levels significantly and independently depended on age, sex, HDL cholesterol, eGFR, and CRP in cross-sectional analyses (**Table 2**), we further adjusted Model 1 for these clinically relevant covariates. In this adjusted Model 2, higher KLK7 serum levels remained to be associated with lower all-cause mortality (adjusted HR 0.64 [95% CI: 0.48-0.84], p = 0.002, **Table 3**), while no significant associations were seen for 4P-MARE or 3P-MACE (**Table 3**). Results did not change when Model 2 was further adjusted for various other known cardio-kidney-metabolic risk factors, such as BMI, systolic blood pressure, ASCVD status, smoking status, LDL cholesterol, and diabetes status (Model 3, **Table 3**). Results were also consistent in sex-stratified analyses, as indicated by non-significant interaction p-values (data not shown).

**Table 3:** Multivariable Cox regression analysis of KLK7 for all-cause mortality, the composite 4-point major adverse renal events (4P-MARE)-endpoint, and the composite 3-point major adverse cardiovascular events (3P-MACE).

Model	HR (95% CI) of KLK7 for all-cause mortality	p	HR (95% CI) of KLK7 for 4P-MARE	p	HR (95% CI) of KLK7 for 3P-MACE	p
Model 1	0.66 (0.53 - 0.84)	**0.001**	1.28 (0.95 - 1.73)	0.108	0.85 (0.63 - 1.16)	0.308
Model 2	0.64 (0.48 - 0.84)	**0.002**	1.06 (0.74 - 1.50)	0.761	1.00 (0.68 - 1.46)	0.980
Model 3	0.65 (0.49 - 0.86)	**0.003**	1.05 (0.74 - 1.50)	0.775	0.98 (0.68 - 1.43)	0.921

The analyses show the adjusted hazard ratios (HR) and 95% confidence intervals (CI) of baseline KLK7 (Log2 transformed before analysis). Adjusted HR are expressed per one unit difference in the log2-transformed KLK7 concentration, equivalent to a doubling of serum KLK7. Participants, who were already on dialysis therapy at baseline, were excluded for the 4P-MARE analysis.

Model 1 was not adjusted.

Model 2 was adjusted for age and sex, HDL cholesterol, eGFR, and CRP.

Model 3 included all variables in Model 2 plus BMI, SBP, atherosclerotic cardiovascular disease status, smoking status, LDL cholesterol, and diabetes status.

Abbreviations as indicated in **Table 1**.Bold markers indicate statistically significant p values (p<0.05).

**Figure 1:**
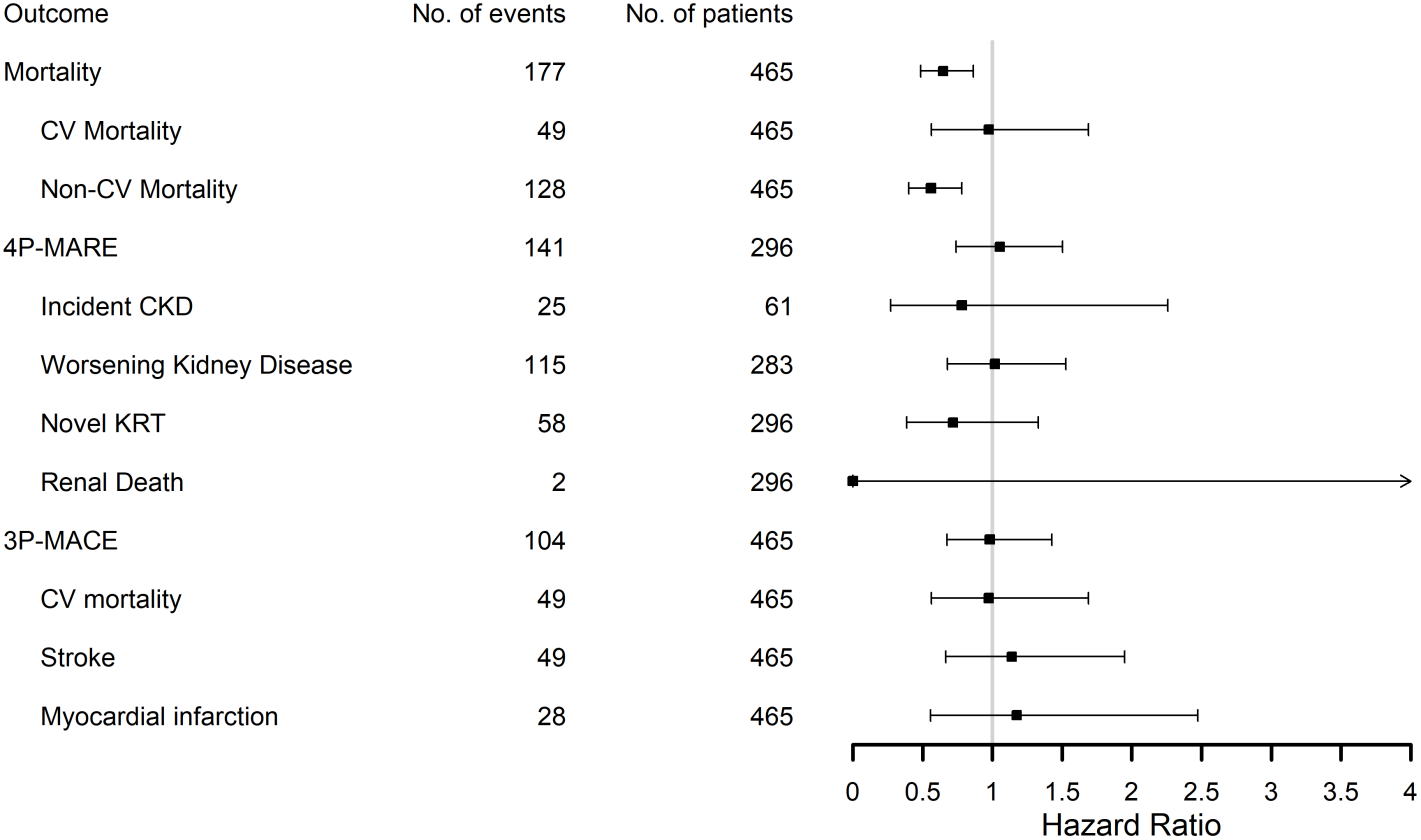
Forest plot of all-cause mortality, 4P-MARE, and 3P-MACE and respective sub-events. Hazard Ratios and 95% confidence intervals for Cox regression Model 3 are shown. Upper confidence interval of renal death exceeds plots limits. Participants, who were already on kidney replacement therapy (KRT) at baseline, were excluded for the 4P-MARE analysis and participants, who already had chronic kidney disease (CKD) at baseline, were excluded for the incident CKD analysis. Number of events, as well as number of patients for each endpoint, is given, respectively. 3P-MACE, 3-point major adverse cardiovascular events; 4P-MARE, 4-point major adverse renal events; CV, Cardiovascular; No., Number.

When analyzing the individual components of the composite outcomes, KLK7 was significantly associated with non-CV mortality (adjusted HR 0.56 [0.40-0.78], p < 0.001), but not with CV mortality (**Figure 1**). No specific subevents of 4P-MARE or 3P-MACE showed a significant association with KLK7.

### Continuous risk prediction and patient risk stratification

**Figure 2:**
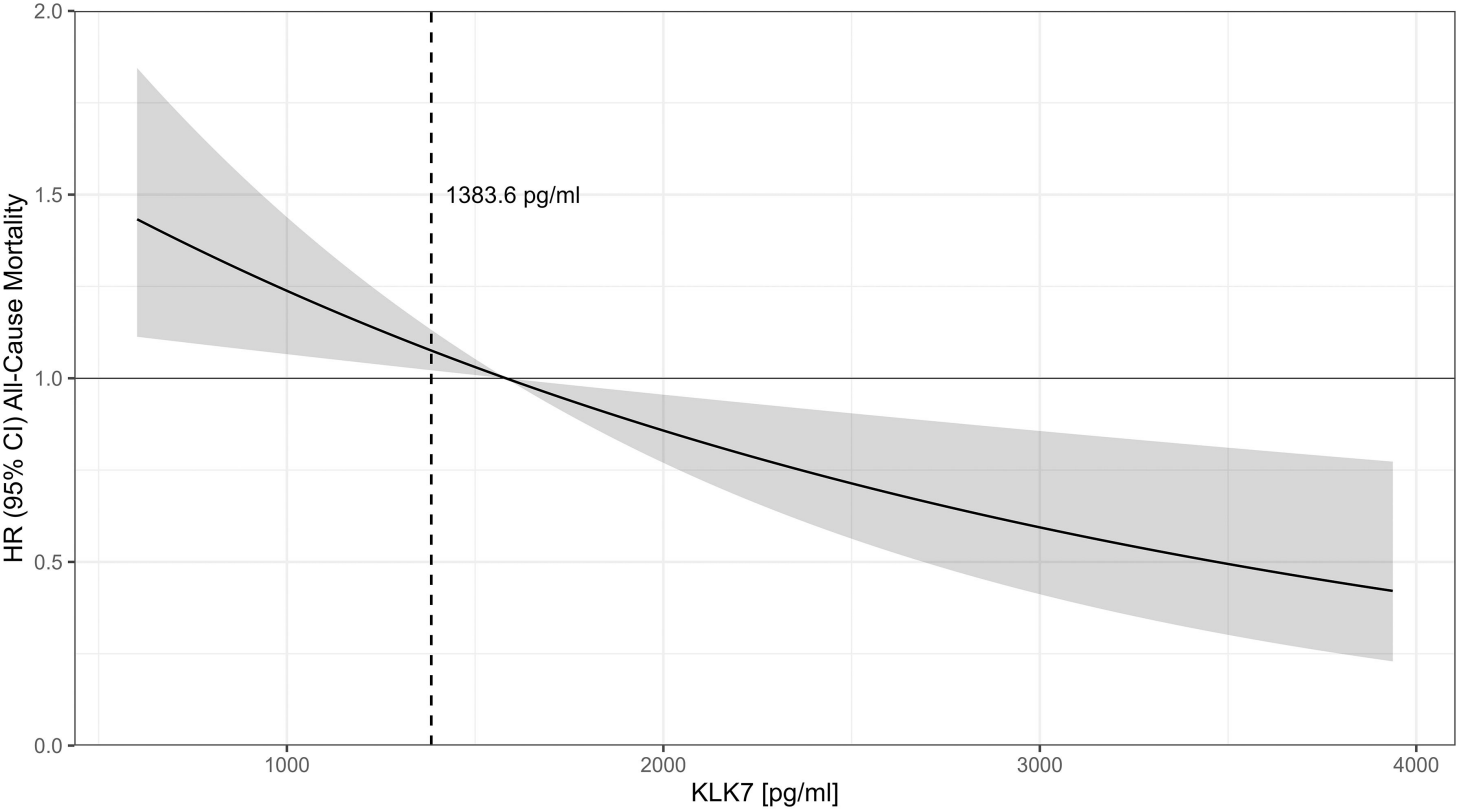
KLK7 concentration-dependent Hazard ratios (HR) for all-cause mortality. The black line shows the continuous HR across the range of circulating KLK7 and the grey-shaded area represents the 95% Confidence Intervals (95% CI) based on Cox regression Model 3 (**Table 3**). Dashed black line indicates the optimal cut-off point (1383.6 pg/ml) as determined by Youden’s Index.

When examining the continuous association between KLK7 and all-cause mortality across its entire range, adjusted HRs visually appeared to cross the line of unity at approximately 1550 pg/ml (**Figure 2**). To derive a data-driven threshold for patient risk stratification, Youden’s Index was applied to a ROC analysis of the fully adjusted Model 3, yielding an optimal cut-off value of 1383.6 pg/ml to differentiate between high- and low-risk individuals for all-cause mortality (**Figure 2**).

### Bulk mRNA sequencing in mice with CKD compared to non-CKD control animals (≥5 mice/group)

**Figure 3:**
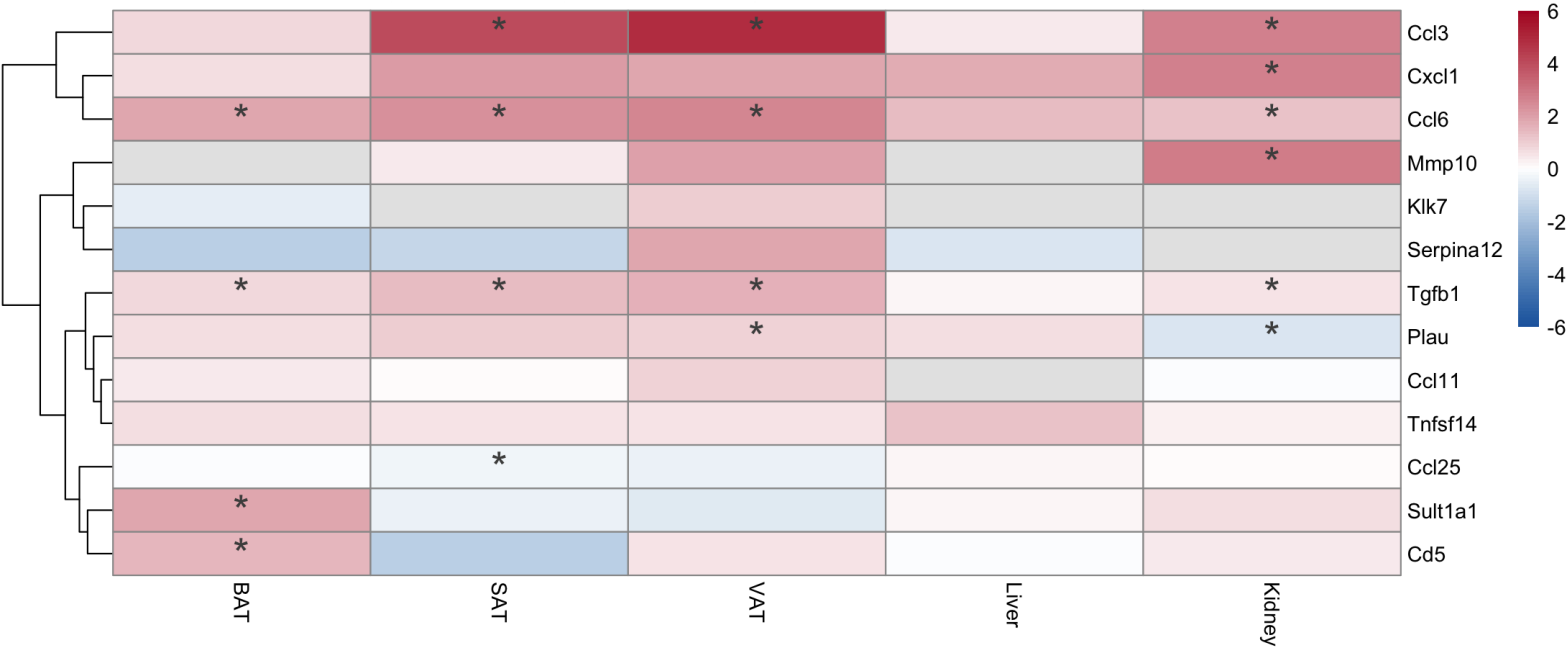
Differential expression analysis of mice with chronic kidney disease (CKD) compared to non-CKD littermate controls in five mouse tissues. Log2 fold changes of mRNA expression of Klk7, Serpina12, and other genes that have previously been related to KLK7 serum levels and adipose tissue *KLK7* mRNA expression in patients with obesity [11] are depicted. Color indicates Log2 fold change. Genes in grey are not expressed in the respective tissue. *indicates statistical significance between mice with CKD and non-CKD control animals (adjusted p-value <0.05). BAT, Brown adipose tissue; SAT, Subcutaneous adipose tissue; VAT, Visceral adipose tissue.

To assess mRNA expression of *Klk7* and related genes in CKD, bulk RNA-seq data from db/db;eNOS^-/-^ (CKD) compared to eNOS^-/-^ (control) mice was analyzed from VAT, SAT, and BAT, as well as kidney and liver. *Klk7* expression was low and detectable only in VAT and BAT, with no significant difference between groups (**Figure 3**). We then analyzed other mRNA expression of genes previously linked to KLK7 in patients with obesity (11). *Serpina12* (vaspin), a key KLK7 inhibitor (8), also did not show differential expression between CKD and non-CKD mice. *Tgfb1* and *Ccl6* were upregulated in CKD mice in all tissues except liver. Most selected genes were differentially expressed in kidney (6/13), while none changed in liver, indicating increased pro-inflammatory gene expression in CKD mice (**Figure 3**). Finally, *Klk7* expression in VAT did not show a significant correlation with any of these genes (**Supplementary Table 3**).

## Discussion

In our CKD cohort, we show that KLK7 is strongly and independently associated with markers of renal function and inflammation in cross-sectional analyses. Furthermore, higher baseline serum levels of KLK7 are an independent predictor of lower all-cause mortality, while no associations have been observed with 4P-MARE or 3P-MACE. In our murine data, *Klk7* mRNA expression does not differ between CKD and non-CKD animals in VAT and BAT, whereas *Klk7* mRNA expression cannot be detected in kidneys, liver, and SAT.

Independent and inverse associations of KLK7 with markers of renal function in cross-sectional analyses are in line with other studies on different metabolic cytokines, such as follistatin-like 3 (15), progranulin (30, 31), proenkephalin (14), proneurotensin (17), acyl-CoA-binding protein (16). In our CKD mouse model, *Klk7* mRNA expression was uniformly low across all examined tissues and undetectable in the kidneys, liver and SAT. In VAT and BAT, where expression could be detected, *Klk7* mRNA was not upregulated in CKD mice compared to non-CKD control mice. Therefore, our murine data suggests that *Klk7* mRNA expression changes in the investigated tissues do not account for elevated KLK7 levels in patients with CKD. Based on these results and KLK7’s relatively low molecular weight of ~26kDa (32), a reduced renal clearance of KLK7 with declining kidney function appears plausible for our observed results. This has also been shown for many small molecular weight proteins such as B-type natriuretic peptide and amino terminal pro-B-type natriuretic peptide (33). Furthermore, and in line with our murine results, Woroniecka and colleagues did also not observe an increase in *KLK7* expression in human glomeruli from patients with diabetes and CKD as compared to healthy controls (12). Analyses from other independent renal−transcriptome datasets, available through the *Nephroseq* database (34), likewise did not reveal any upregulation of *KLK7* in human kidney tissue samples from a variety of other CKD etiologies (35–37). Thus, although species−specific differences in *KLK7* regulation cannot be completely ruled out, neither our murine experiments nor the examined human transcriptomic cohorts show evidence of *KLK7* upregulation in the context of CKD. (38–40) However, *KLK7* is also expressed in tissues beyond the kidney, including esophagus, skin, bone marrow, and female reproductive tissues (41). Therefore, increased circulating KLK7 levels observed in our CKD cohort may, at least in part, originate from other sources. Future studies employing further tissue-specific analyses or single-cell approaches need to identify the sources of circulating KLK7 in CKD more precisely. Notably, *KLK7* expression is markedly upregulated in inflammatory skin conditions, such as atopic dermatitis (6, 7). Moreover, patients with CKD more frequently present with common inflammatory skin diseases, including atopic eczema and psoriasis (42) compared with individuals without CKD. Thus, inflammatory skin diseases may represent a confounding factor in our analysis and could potentially account for the elevated serum KLK7 levels observed in our cohort. However, adjustment for this factor was not feasible because skin conditions were not systematically assessed in this study population. Furthermore, age-related changes of the skin, including stratum corneum (43), may further influence KLK7 levels and thereby affect our cross-sectional associations although our full models have been adjusted for age.

Chronic inflammation has emerged as a pivotal non-traditional risk factor in the progression of CKD, contributing significantly to morbidity and mortality in this population (2, 44). (11) KLK7 has been linked to obesity−related inflammatory pathways (11). Given its inverse cross-sectional associations with eGFR and inflammation, we assessed its prognostic relevance in CKD. While KLK7 did not predict 4P−MARE or 3P−MACE, higher baseline levels were independently associated with lower all−cause mortality. To our knowledge, this is the first demonstration of a protective KLK7-mortality association in a CKD cohort. These seemingly contradictory results between lower eGFR cross-sectionally and lower mortality prospectively may reflect the pattern of “reverse epidemiology” in advanced CKD, where biomarkers and/or traditional risk factors in advanced CKD show paradoxical associations with outcomes (45–47), supported by the inverse relationship between KLK7 and CRP presented here. Context-dependent effects of kallikreins may also contribute: KLK1, for example, can promote inflammation yet protect against DKD by reducing oxidative stress and fibrosis (48), underscoring complex, tissue−specific functions within the kallikrein family. Alternatively, elevated KLK7 levels in lower eGFR categories may represent a compensatory response conferring protection against non-renal causes of death, particularly infections and malignancies, as suggested by our finding that KLK7 predicted non-CV, but not CV or renal mortality.

Supporting this, tissue expression of *KLK7* has been previously associated with survival in distinct cancer types. Thus, high *KLK7* expression in ovarian cancer tissue has been linked to reduced death and relapse risk (49). One study showed a borderline inverse association of *KLK7* mRNA expression in ovarian cancer tissue with overall survival in univariable Cox regression analysis (50). However, conflicting data have also been presented (51, 52). However, while we confirm *Klk7* mRNA expression in VAT and BAT samples in our CKD mice, murine *Klk7* expression in VAT does not correlate with other genes that have previously been related to KLK7 serum levels and *KLK7* adipose tissue mRNA expression in patients with obesity (11), including several pro-inflammatory genes (**Supplementary Tables 1**, **3**). Future studies, therefore, need to investigate whether KLK7 has a direct anti-inflammatory role in CKD by which it potentially can affect mortality.

Some limitations of this study should be acknowledged. First, the generalizability of our findings to other CKD cohorts is limited, as the cohort primarily comprised Caucasian participants from Saxony, Germany. Second, despite high follow-up rates, loss to follow-up may have introduced selection bias. Third, only *Klk7* mRNA expression was assessed, whereas protein-level measurements (e.g., by Western blot or immunohistochemistry) could provide a more accurate reflection of circulating KLK7 in our mouse experiments, as mRNA expression of proteases does not necessarily correspond to their protein abundance or activity. Finally, the CKD mouse model used in this study may not fully recapitulate human disease, and alternative CKD models might yield different patterns of *Klk7* expression.

## Conclusion

In our CKD cohort, KLK7 serum levels are independently associated with declining kidney function, potentially through reduced KLK7 clearance. Higher circulating KLK7 independently predicts lower all-cause mortality with an optimal cut-off point at 1383.6 pg/ml, whereas baseline KLK7 was not related to composite renal and CV outcomes.

## Data Availability

Raw data cannot be shared publicly because of consent restrictions of the included clinical cohort. Data sets used and/or analyzed during the current study are available from the corresponding author on reasonable request.
