# Supplementary material for: Inverse association of circulating kallikrein-related peptidase 7 with renal function and mortality risk in patients with chronic kidney disease
Source: Front Endocrinol (Lausanne). 2026 Jul 7;17:1804956. doi: 10.3389/fendo.2026.1804956 (PMC13384942; doi:10.3389/fendo.2026.1804956)
Supplement: Supplementary file 1 [file Table1.pdf]

Supplementary Table S1: Univariate correlation analyses and multivariable linear regression analysis of serum KLK7 with anthropometric parameters and markers of glucose metabolism, serum lipids, inflammation, and renal function for female individuals.

	**Univariate correlation analyses**		**Multivariable linear regression analysis**	
	**r**	**p**	**β**	**p**
Age (years)	**-0.143**	**0.029**	**-0.178**	**0.009**
Sex	-	-	-	-
BMI (kg/m^2)	-0.038	n.s.	-	-
Waist circumference (cm)	-0.019	n.s.	-	-
Waist-to-height ratio	-0.046	n.s.	0.124	n.s.
SBP (mmHg)	-0.016	n.s.	-	-
DBP (mmHg)	0.018	n.s.	-	-
HbA1c (%)	0.134	n.s.	-	-
FG (mmol/l)	-0.019	n.s.	-	-
FI (pmol/l)	0.039	n.s.	-	-
Cholesterol (mmol/l)	0.036	n.s.	-	-
HDL cholesterol (mmol/l)	0.010	n.s.	-	-
LDL cholesterol (mmol/l)	0.055	n.s.	-	-
TG (mmol/l)	0.004	n.s.	-	-
Creatinine (µmol/l)	**0.172**	**0.009**	-	-
eGFR (ml/min/1.73m^2)	**-0.145**	**0.027**	**-0.178**	**0.006**
uACR (mg/g)	0.058	n.s.	-	-
CRP (mg/l)	**-0.190**	**0.004**	**-0.318**	**<0.001**

Supplementary Table S1. Univariate correlation analyses and multivariable linear regression analyses of KLK7 with anthropometric and biochemical markers for female individuals. Non-parametric Spearman's rank correlation method was used to assess univariate relationships between KLK7 and indicated markers. Multivariable regression analysis was calculated for KLK7 (lg, dependent variable) adjusted for age (lg), Waist-to-height ratio, eGFR (lg), as well as CRP (lg). Non-normally distributed variables as assessed by Shapiro-Wilk-test were logarithmically transformed prior to multivariable testing (lg). r- and p-values, as well as standardized β-coefficients and p-values, are given. **Bold** markers indicate significant correlations in univariate analysis or independent associations in multivariable analyses. Abbreviations are indicated in Table 1.

Supplementary Table S2: Univariate correlation analyses and multivariable linear regression analysis of serum KLK7 with anthropometric parameters and markers of glucose metabolism, serum lipids, inflammation, and renal function for male individuals.

	**Univariate correlation analyses**		**Multivariable linear regression analysis**	
	**r**	**p**	**β**	**p**
Age (years)	**-0.329**	**<0.001**	**-0.285**	**<0.001**
Sex	**-**	-	**-**	**-**
BMI (kg/m^2)	**-0.153**	**0.007**	-	-
Waist circumference (cm)	**-0.167**	**0.003**	-	-
Waist-to-height ratio	**-0.191**	**<0.001**	-0.014	n.s.
SBP (mmHg)	-0.071	n.s.	-	-
DBP (mmHg)	0.028	n.s.	-	-
HbA1c (%)	**-0.203**	**0.001**	-	-
FG (mmol/l)	**-0.275**	**<0.001**	-0.064	n.s.
FI (pmol/l)	**-0.131**	**0.021**	-0.046	n.s.
Cholesterol (mmol/l)	**-0.115**	**0.043**	-	-
HDL cholesterol (mmol/l)	**-0.153**	**0.007**	**-0.193**	**0.007**
LDL cholesterol (mmol/l)	-0.095	n.s.	-	-
TG (mmol/l)	0.046	n.s.	**-**	**-**
Creatinine (µmol/l)	**0.313**	**<0.001**	**-**	**-**
eGFR (ml/min/1.73m^2)	**-0.277**	**<0.001**	**-0.233**	**0.004**
uACR (mg/g)	**0.161**	**0.019**	0.021	n.s.
CRP (mg/l)	**-0.147**	**0.010**	**-0.205**	**0.004**

Supplementary Table S2. Univariate correlation analyses and multivariable linear regression analyses of KLK7 with anthropometric and biochemical markers for male individuals. Non-parametric Spearman's rank correlation method was used to assess univariate relationships between KLK7 and indicated markers. Multivariable regression analysis was calculated for KLK7 (lg, dependent variable) adjusted for age (lg), BMI (lg), waist circumference (lg), HbA1c (lg), FG (lg), FI (lg), Cholesterol (lg), HDL cholesterol (lg), eGFR (lg), uACR (lg) as well as CRP (lg). Non-normally distributed variables as assessed by Shapiro-Wilk-test were logarithmically transformed prior to multivariable testing (lg). r- and p-values, as well as standardized β-coefficients and p-values, are given. **Bold** markers indicate significant correlations in univariate analysis or independent associations in multivariable analyses. Abbreviations are indicated in Table 1.

Supplementary Table S3: Correlation analysis of *Klk7* mRNA expression with genes of interest in visceral adipose tissue of mice

Gene symbol	Correlation coefficient	p-value	adjusted p-value
Sult1a1	0.253	0.427	0.926
Tnfsf14	-0.424	0.17	0.926
Tgfb1	0.034	0.916	0.926
Cd5	-0.03	0.926	0.926
Ccl6	0.159	0.621	0.926
Cxcl1	0.274	0.39	0.926
Mmp10	0.328	0.298	0.926
Plau	0.219	0.494	0.926
Ccl25	0.064	0.843	0.926
Ccl3	0.133	0.681	0.926
Ccl11	0.393	0.207	0.926
Serpina12	0.032	0.921	0.926

Supplementary Table S3. Correlation of *Klk7* mRNA expression in visceral adipose tissue samples in our mice with mRNA expression levels of genes that have previously been related to KLK7 serum levels and *KLK7* adipose tissue mRNA expression in patients with obesity [12]. The analysis shows correlation coefficients (Pearson), p-values and adjusted p-values (false discovery rate).
